# Factors associated with postnatal care for newborns in Zambia: analysis of the 2013-14 Zambia demographic and health survey

**DOI:** 10.1186/s12884-017-1612-1

**Published:** 2017-12-13

**Authors:** Bupe B. Bwalya, Mulenga C. Mulenga, James N. Mulenga

**Affiliations:** 1 Department of Mathematics and Statistics, School Sciences, Engineering and Technology, Mulungushi University, Town Campus, P.O. Box 80415, Kabwe, Zambia; 2 Department of Economics, School of Social Science, Mulungushi University, Kabwe, Zambia

**Keywords:** Postnatal care, Timing of first postnatal check-up, Newborn, Demographic and socio-economic factors, Home births, Facility births, Zambia

## Abstract

**Background:**

The importance of postnatal care cannot be overemphasised. Various studies undertaken worldwide have found that PNC is critical for the survival of newborns. However, in Zambia, despite much emphasis by the government and various international Organisations on the need for PNC, coverage continues to be low. This study attempted to assess the demographic and socio-economic factors associated with newborns' receipt of PNC and the timing of first PNC in Zambia.

**Methods:**

Based on data from the 2013-14 Zambia Demographic and Health Survey (ZDHS), this study used bivariate, stepwise binary and multinomial logistic regression analyses to examine PNC for births at home and at health facilities.

**Results:**

The results indicate that different factors influence the utilisation of PNC among home births, these include: place of delivery, mothers’ exposure or access to media and having 4+ ANC visits. On the other hand, place of residence and mothers’ access or exposure to media were found to be the determinants of PNC among facility deliveries. The results further indicate that among the home births, mothers’ media exposure or access to media, having secondary or higher education, and having 4+ ANC visits during pregnancy increased the odds of having PNC within 48 hours. Furthermore, attending the first PNC 48 hours after delivery was determined by place of residence, media exposure and 4+ ANC visits. On the other hand, among the facility births, the timing of PNC within 48 hours, was influenced by the perceived size at birth of the newborn.

**Conclusion:**

The study makes the following recommendations: more attention to be given to rural based women and newborns; encourage delivery at health facilities; more emphasis on the importance of ANC visits; and need to disseminate information through various media on the importance of PNC even in rural communities.

## Background

Worldwide, more than one million babies die on their first day of life each year, making the day of birth the most dangerous day for babies in nearly every country [1–3]. Almost all newborn deaths occur in developing countries; with the highest number in South Asia and the highest newborn mortality rates in Sub-Saharan Africa (SSA) [1]. Most of the newborn deaths in SSA occur among children delivered at home or outside a health facility [3]. These deaths are mainly as a result of poor maternal health, inadequate care during pregnancy, inappropriate management of complications during pregnancy & after delivery, poor hygiene during delivery & the first critical hours after birth, and lack of newborn care [4, 5].

Elsewhere, literature reveals that, most of the factors that lead to neonatal deaths could be averted through postnatal check-ups [6–8]. Postnatal care (PNC) is defined as the care given to the newborn baby immediately after birth (within 24 hours) and for the first 6 weeks (42 days) of life, with the aim of ensuring optimum health for the newborn [1, 9–11]. The care received in PNC includes; providing care, monitoring danger signs in the newborns’ breathing, temperature, breastfeeding, and movement as well as counselling the mother on health, nutrition, and healthy lifestyle practices [3, 12, 13].

The World Health Organisation (WHO) recommendations on PNC prescribe that, for every uncomplicated vaginal birth in a health facility, healthy newborns should receive care in the facility for at least 24 hours. In case the birth occurs at home, the first postnatal contact should be at least within 24 hours of birth. Regardless of place of delivery, at least three additional postnatal contacts are recommended for all mothers and newborns, on day 3 (48-72 hours), between days 7 and 14, and 6 weeks after birth [14]. However, less than a quarter of newborns in less developed countries receive PNC within 48 hours of delivery [3, 13].

The 2013-14 Zambia Demographic Health Survey (ZDHS) shows some improvement in the survival rates of infants and of children under age 5 in recent years. Statistics show that the under-five mortality rate dropped from 128 deaths per 1,000 live births in 2003 to 75 deaths per 1,000 live births in 2013-14, and infant mortality declined from 76 deaths per 1,000 live births in 2003 to 45 deaths per 1,000 live births in 2013-14. In spite the fact that, nearly two-thirds of deliveries occur in health facilities, there have been only marginal improvements in neonatal mortality (NNM) in the past 10 to 15 years, from 29 deaths per 1,000 live births between 1999 and 2003 to 24 deaths per 1,000 live births between 2009 and 2013. This scenario may be explained in part by the low level of PNC (16%). Moreover, one-third of home delivered newborns, are even less likely to receive PNC within the first 2 days (8%) than those delivered in a health facility (19%) [15]. This is despite the fact that the Government of the Republic of Zambia through the Ministry of Health (MoH) and Ministry of Community Development Mother and Child Health (MCDMCH) and other cooperating partners have developed and put in place various policies, programs, and interventions aimed at improving the general welfare and health of children.

The Zambian government has been committed to the promotion of child health through its policy and program implementation, some of which include: Integrated Community Case Management of Childhood Illnesses (ICMCI), with the support from UNICEF and other partners, the government has introduced integrated case management of pneumonia, malaria, diarrhoea, and malnutrition in 23 selected districts. The government intends to scale it up to all districts of the country. To date, 1 209 community health workers have been trained. In addition, in order for the government to improve safe motherhood and newborn health, the government through the Ministry of Health has provided mentorship to six of the ten provinces of the country. District teams have also been trained on emergency obstetric and newborn care (EmONC) and safe motherhood action groups (SMAGs) have been established, trained and provided with basic supplies in an effort to reach the highly disadvantaged populations in urban and rural areas [16]. Expanded Programme on Immunization (EPI) is also another program government has been able to implement. The program is aimed at scaling up and sustaining high-impact nutrition interventions, including early initiation of breastfeeding, among others [17]. All these programs aim at reducing the rates and levels of neonatal and post-neonatal mortality in Zambia.

In Zambia, studies related to PNC among newborns and based on the nationally representative surveys like the ZDHS are scarce. This is because data on PNC among newborns had never been collected until recently during the 2013-14 ZDHS. Despite this dearth in empirical literature in Zambia, studies in other countries have shown that, regardless of whether delivery was at home or in a health facility, several factors contribute to low levels of PNC, including mother’s age at birth [12], perceived size at birth [3, 8], maternal education [6, 12], household wealth [6, 8, 12, 18], maternal employment status [12], geographic distance, such as household distance to a health facility [6, 8], place of delivery, lack of antenatal care, and place of residence, among others [6, 8, 19–22].

To increase coverage of PNC in Zambia, a better understanding of its associated factors is important. The objectives of this study were twofold: first, to assess the demographic and socioeconomic factors associated with any PNC for newborns; and second, to examine the demographic and socioeconomic factors associated with the timing of the first PNC. It is envisaged that an understanding of such factors may help develop necessary strategies and interventions to help improve PNC coverage and in turn improve neonatal survival in Zambia.

### Conceptual framework

**Fig. 1:**
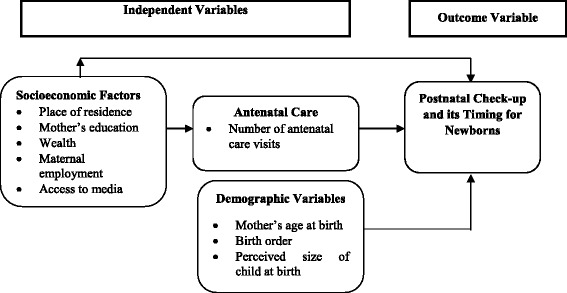
Conceptual Framework

The conceptual framework below (Fig. 1) shows the linkages expected between PNC for newborns and demographic, socioeconomic, and ANC factors. The variables used in this conceptual framework were based on the empirical evidence from other similar studies and also their availability in the 2013-14 ZDHS. As the framework indicates, there could be direct links between socioeconomic factors and PNCs for newborns. For instance, place of residence (urban or rural) is an important factors as it may affect PNC coverage. The reasoning here is that; urban residents tend to have higher PNC coverage due to the availability of more health facilities compared to rural areas. Mothers’ educational level, wealth, and employment status could be positively associated with newborns receipt of PNC. Mothers with more education, wealth, and employment may have better understanding of the importance of PNC to their newborns’ survival. This, coupled with better access to media, may broaden women’s knowledge of how access to PNC can improve the health status and survival of newborn children.

Further, demographic variables may also have some influence on a child’s receipt of PNC. For example, first-order births and newborns whose mothers made the recommended four or more ANC visits may be more likely to receive PNC.

## Methods

### Data

This study used data for women from the 2013-14 ZDHS to assess the demographic and socioeconomic factors associated with postnatal care for newborns. The ZDHS used a two-stage cluster sampling method. In the first stage, 722 enumeration areas (EAs)—305 in urban areas and 417 in rural areas-were selected with probability proportional to Sample Enumeration Area (SEA) size. In the second stage, a complete list of households served as the sampling frame in the selection of households for enumeration. On average, 25 households were randomly selected in each cluster. All women age 15-49 in the household were eligible to be interviewed. Women were asked to provide information about pregnancies resulting in a live birth during the 5 years preceding the interview. In this case, a live birth is defined as “the complete expulsion or extraction from its mother of a product of conception, irrespective of the duration of pregnancy, which after such separation, breathes or shows any other evidence of life, such as beating of the heart, pulsation of the umbilical cord, or any definite movement of voluntary muscles, whether or not the umbilical cord has been cut or the placenta is attached” [23]. Data on postnatal care was collected for the most recent birth during that period.

In the 722 selected clusters, 16,258 households were occupied at the time of data collection of which 15,920 were successfully interviewed, yielding a household response rate of 98%. In total, 16,411 women were successfully interviewed. As observed from above, there were more women who were interviewed than the number of households sampled because once interviewers visited the household, they interviewed every woman in the household so long they were aged 15 – 49 years. This analysis concentrated on the postnatal care received for the woman’s most recent birth in the 5 years preceding the survey. Among the most recent births, 514 caesarean births were excluded from the analysis sample because these births were likely to have received PNC regardless of the mothers’ demographic and socioeconomic characteristics. The final sample included 4,777 births, with 1,361 delivered at home and 3,416 delivered in a health facility.

### Outcome and explanatory variables used in the analysis

In the DHS Woman’s Questionnaire, all women who had a birth in the 5 years preceding the survey were asked this question about their most recent birth: *“How long after delivery did the first check take place for last birth?”* Responses to this question were used to construct the two outcome variables for this study. These variables were any postnatal check-up within 6 weeks after delivery “0 = No PNC; and 1=Yes any PNC” and timing of first PNC “0 = No PNC”, “1 > 48 hours”, and “2 ≤ 48 hours” 2 or more days [24]. The categorization of timing was done in this way because most neonatal deaths usually occur within the 48 hours after the child’s birth [25]. The rationale behind using the two measures was based on the fact that not only is receiving any PNC cardinal but also receiving it within the most critical period (within 48 hours) can increase the chances of survival for the newborn.

Demographic variables included in the model were mother’s age at the last birth (under age 20, 20-34, and 35-49), birth order (1st, 2nd, 3rd, and 4th or higher), and mother’s perception of the size of the child (small, average, or large).

Among the socioeconomic factors, we examined mother’s employment status (whether currently working or not), education attainment (none, primary, and secondary or higher), and household wealth status. The DHS wealth quintiles were grouped into a three-category wealth status variable (poor, middle, and rich) by combining the top 40% and the bottom 40% of the population. Media access was constructed on a combination of three variables (frequency of reading newspapers or magazines, listening to radio, and watching television). A mother is considered to have access to mass media if she watches TV, listens to radio, or reads a newspaper. Receipt of antenatal care was measured with the number of ANC visits the mother made for the last birth (0 = less than four, 1 = four or more).

### Statistical analysis

Separate analyses were conducted for home deliveries and facility deliveries because the factors that influence PNC for newborns delivered at home may be different from those that influence newborns delivered in a health facility [3, 26, 27].

Descriptive statistics (both univariate and bivariate) were used to describe the overall coverage of PNC and timing of PNC, and by demographic and socioeconomic variables, as well as by the number of ANC visits received.

To assess the adjusted associations between the independent variables and receipt of any PNC for newborns, two models were fitted, one for newborns delivered at home and the other for those delivered in a health facility. Odds ratios with 95% confidence intervals were reported.

In addition, a stepwise backward regression was used to assess a newborn receiving or not receiving PNC and timing of PNC with their associated correlates stratified by place of delivery. In both models, “never received any PNC” was the reference category. All the estimates were weighted to reflect the population. The effect of the complex multistage sampling design that was used in the 2013-14 ZDHS was considered. Data were analysed using STATA 14.2 software.

## Results

### Description of the analysis sample

Table 1 below describes births included in the analysis by mother’s characteristics. The total sample for the analysis was 4,777 newborns of which 3,416 (71.5%) were delivered at health facilities while 1,361 (28.5%) were delivered at home. For both home and facility births, about two-thirds of the mothers were aged 20-34 at the time of birth. Newborns delivered at home had a higher proportion of 4th order births (60%) than newborns delivered in a health facility (40%). Less than two-thirds of newborns were perceived by their mothers to have average size at birth (61% delivered at home and 57% delivered in a facility).

**Table 1:** Percent distribution of the sample by demographic, socioeconomic, antenatal care services according to place of delivery, 2013-14 ZDHS

Variable	Home		Facility		Total	
	%	n	%	n	%	n
Mother’s Age at Birth						
<20	13.4	183	20.3	694	18.3	877
20-34	66.0	899	66.9	2,286	66.7	3,187
35+	20.6	280	12.8	436	15.0	715
Birth Order						
1^st^	10.5	143	25.2	860	21.0	1,003
2^nd^	15.3	208	18.7	640	17.7	847
3^rd^	14.6	199	16.4	561	15.9	761
4th or more	59.6	811	39.7	1,355	45.4	2,168
Perceived Size at Birth						
Small	13.8	188	9.8	336	11.0	524
Average	60.7	827	57.0	1,946	58.0	2,772
Large	25.5	347	33.2	1,134	31.0	1,483
Region						
Central	18.2	248	7.3	248	10.4	496
Copperbelt	6.5	88	14.9	508	12.5	596
Eastern	10.1	138	13.8	472	12.8	610
Luapula	9.0	123	9.1	310	9.0	432
Lusaka	3.2	44	18.9	646	14.4	689
Muchinga	7.0	95	5.7	196	6.1	291
Northern	14.5	197	6.9	236	9.1	434
North Western	3.7	50	5.4	184	4.9	234
Southern	18.7	255	12.0	410	14.0	667
Western	9.0	123	6.0	206	6.9	329
Type of Place of Residence						
Urban	9.5	130	42.3	1,445	33.0	1,575
Rural	90.5	1,231	57.7	1,971	67.0	3,204
Highest Educational Level						
None	16.7	227	8.5	290	10.8	517
Primary	65.9	895	50.1	1,711	54.6	2,608
Secondary+	17.3	235	41.4	1,415	34.6	1,650
Wealth Index						
Poor	69.5	946	39.7	1,355	48.2	2,304
Middle	21.7	296	20.3	695	20.7	990
Rich	8.8	119	40.0	1,365	31.1	1,485
Mother’s Employment Status						
Unemployed	37.1	504	46.8	1,593	44.0	2,099
Employed	62.9	854	53.2	1,813	56.0	2,667
Media Access						
No access	45.8	623	27.1	925	32.4	1,550
Access to media	54.2	739	72.9	2,491	67.6	3,229
Number of ANC visits						
<4	54.1	736	44.6	1,523	47.3	2,261
4+	45.9	625	55.4	1,893	52.7	2,518
Total	100	1,361	100	3,416	100	4,777

By geographic distribution, a larger share of home births took place in Central and Southern regions (18% and 19%), while Eastern, Copperbelt, and Lusaka regions had a larger share of facility births (14%, 15%, and 19%). In every 10 home deliveries 9 were in rural areas (91%) compared with about 6 in every 10 deliveries in a health facility (58%). A majority of mothers had primary education, whether they delivered at home or in a health facility (66% and 50%). Among newborns delivered at home, more than two-thirds were in poor households, while among those delivered at a health facility, poor and rich households accounted for about 40% each. Sixty-three percent of mothers of newborns delivered at home were employed at the time of the survey, as were 53% of mothers of newborns delivered in a health facility. Nearly three-fourths (73%) of the mothers of newborns delivered in a health facility had access to media compared with about half (54%) of mothers of newborns delivered at home.

Overall, more than half (53%) of the mothers of the newborns had four or more ANC visits. Disaggregated by place of delivery, 55% of mothers of newborns delivered in a health facility had four or more ANC (4+ ANC) visits compared with 46% among those delivered at home.

### Coverage of postnatal care and timing of the first PNC for newborns

**Fig. 2:**
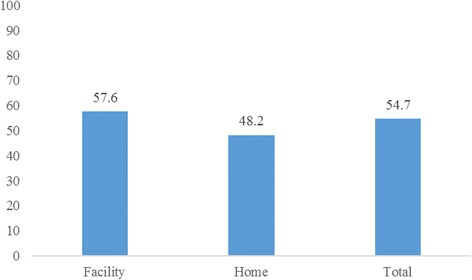
Percentage of newborns who received any postnatal care, by place of delivery

Figure 2 below shows the percentage of newborns who received PNC after birth by place of delivery. Overall, 55% of the newborns received PNC after birth. Disaggregated by place of delivery, 58% of newborns delivered in a health facility received PNC compared with less than half (48%) of newborns delivered at home.

**Fig. 3:**
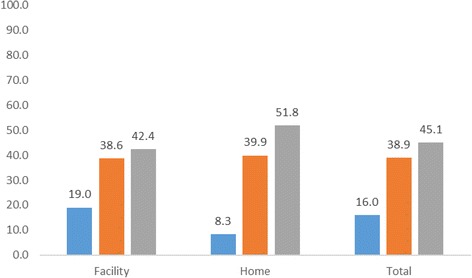
Percent distribution of newborns by timing of the first postnatal check-up

Figure 3 below shows the percent distribution of timing of first PNC after birth by place of delivery. Overall, only 16% of newborns received their first PNC within the first 2 days. Among newborns delivered in a health facility, 19% received their first PNC within the first 2 days compared with 8% among newborns delivered at home.

### Bivariate analysis results of postnatal care for newborns

Table 2 below shows the percentage of newborns who received PNC after birth according to demographic and socioeconomic characteristics. Overall, PNC coverage varied significantly across place of residence, with newborns delivered at health facilities in rural areas less likely to receive PNC than newborns delivered at health facilities in urban areas (55% versus 62%). In addition, PNC coverage varied significantly by whether mothers of newborns had access to media. Among newborns whose mothers had access to media, a higher proportion received PNC compared with newborns whose mothers did not have access to media—at 53% versus 42% for home deliveries and 590% versus 53% for deliveries in a health facility.

**Table 2:** Percentage of newborns who received any postnatal checkups after birth, according to demographic and socioeconomic characteristics by place of delivery, 2013-14 ZDHS

Variable	Home births			Facility births		
	%	CI	*p*-values	%	CI	*p*-values
Mother’s Age at birth						
<20	41.9	[33.9,50.4]	0.226	55.7	[50.9,60.5]	0.430
20-34	49.6	[45.8,53.5]		58.5	[55.3,61.7]	
35+	47.7	[40.8,54.6]		55.8	[49.6,61.8]	
Birth Order						
1^st^	49.2	[40.5,57.9]	0.843	57.6	[53.0,62.0]	0.985
2^nd^	45.0	[37.2,53.0]		58.2	[53.0,63.2]	
3^rd^	49.4	[41.7,57.1]		58.1	[52.7,63.3]	
4th or more	48.5	[44.0,53.1]		57.2	[53.4,61.0]	
Perceived Size at Birth						
Small	42.6	[35.0,50.6]	0.209	56.8	[50.6,62.8]	0.906
Average	50.4	[45.7,55.1]		57.4	[54.1,60.7]	
Large	45.9	[39.4,52.5]		58.2	[53.6,62.7]	
Type of Residence						
Urban	56.7	[47.9,65.1]	0.052	61.7	[56.6,66.6]	0.023
Rural	47.3	[43.6,51.0]		54.6	[51.2,58.0]	
Highest Educational Level						
None	48.0	[40.8,55.3]	0.412	61.1	[54.4,67.3]	0.293
Primary	47.1	[42.8,51.4]		58.6	[55.2,61.9]	
Secondary +	52.6	[45.5,59.6]		55.8	[51.3,60.1]	
Wealth Index						
Poor	47.7	[43.7,51.7]	0.748	55.9	[52.5,59.4]	0.200
Middle	50.5	[42.8,58.2]		55.5	[50.3,60.6]	
Rich	46.6	[36.9,56.6]		60.4	[55.2,65.3]	
Womens Employment Status						
Unemployed	49.1	[43.0,55.3]	0.685	58.9	[54.6,63.0]	0.372
Employed	47.5	[43.0,52.0]		56.7	[53.3,60.0]	
Media Exposure						
No access	42.0	[37.2,47.1]	0.001	53.2	[49.1,57.2]	0.013
Access to media	53.4	[48.7,58.0]		59.3	[55.8,62.6]	
ANC visits						
<4	45.5	[41.2,50.0]	0.065	57.4	[53.7,61.0]	0.070
>=4+	51.3	[46.5,56.0]		57.8	[54.2,61.3]	
Total	48.2	[44.8,51.7]		57.6	[55.1,60.8]	

### Bivariate analysis results of timing of the first postnatal check-ups

Table 3 below shows the percentage of newborns by timing of first PNC according to demographic and socioeconomic characteristics. Among newborns delivered at home, the proportion receiving their first PNC within the first 48 hours after birth was twice as high in urban areas as in rural areas (15% versus 8%). For facility deliveries, newborns in urban areas had a slightly higher proportion of receiving their first PNC within the first 48 hours compared with those in rural areas (41% versus 38%).

**Table 3:** Percent distribution of newborns delivered at home and in a health facility by timing of the postnatal checkups according to demographic and socioeconomic characteristics, 2013-14 ZDHS

Variable	Home births							Facility births						
	None		Within 48 hours		After 48 hours		*p*-value	None		Within 48 hours		After 48 hours		*p*-value
	%	CI	%	CI	%	CI		%	CI	%	CI	%	CI	
Mother’s age at birth														
<20	58.1	[49.6,66.1]	5.8	[3.2,10.2]	36.1	[28.4,44.5]	0.283	44.3	[39.5,49.1]	18.7	[15.3,22.8]	37.0	[32.6,41.6]	0.682
20-34	50.4	[46.5,54.2]	9.3	[7.3,11.8]	40.3	[36.8,44.0]		41.5	[38.3,44.7]	19.5	[17.4,21.9]	39.0	[36.0,42.1]	
35+	52.3	[45.4,59.2]	6.6	[4.0,10.8]	41.0	[34.7,47.7]		44.2	[38.2,50.4]	17.0	[13.1,22.0]	38.7	[32.9,44.8]	
Birth Order														
1st	50.8	[42.1,59.5]	10.4	[5.9,17.7]	38.8	[30.7,47.5]	0.911	42.4	[38.0,47.0]	21.4	[17.9,25.3]	36.2	[32.2,40.4]	0.596
2nd	55.0	[47.0,62.8]	6.4	[3.6,11.2]	38.6	[31.0,46.7]		41.8	[36.8,47.0]	19.9	[16.4,24.0]	38.2	[33.4,43.4]	
3rd	50.6	[42.9,58.3]	9.2	[5.7,14.6]	40.2	[33.0,47.8]		41.9	[36.7,47.3]	17.5	[13.6,22.3]	40.6	[35.1,46.3]	
4th or more	51.5	[46.9,56.0]	8.1	[6.3,10.5]	40.4	[36.1,44.9]		42.8	[39.0,46.6]	17.8	[15.3,20.6]	39.4	[35.9,43.0]	
Perceived Size at Birth														
Small	57.4	[49.4,65.0]	5.3	[2.6,10.5]	37.3	[29.9,45.3]	0.375	43.2	[37.2,49.4]	25.8	[20.2,32.3]	31.0	[25.5,37.2]	0.069
Average	49.6	[44.9,54.3]	9.0	[6.9,11.6]	41.4	[37.1,45.8]		42.6	[39.3,45.9]	17.8	[15.5,20.5]	39.6	[36.6,42.7]	
Large	54.1	[47.5,60.6]	8.1	[5.4,12.1]	37.8	[31.9,44.0]		41.8	[37.3,46.4]	19.1	[15.8,22.9]	39.1	[34.5,43.9]	
Type of Residence														
Urban	43.3	[34.9,52.1]	15.0	[8.9,24.1]	41.7	[33.7,50.2]	0.019	38.3	[33.4,43.4]	21.2	[17.8,25.1]	40.5	[36.1,45.1]	0.041
Rural	52.7	[49.0,56.4]	7.6	[6.0,9.5]	39.7	[36.3,43.2]		45.4	[42.0,48.8]	17.5	[15.2,20.0]	37.2	[33.8,40.6]	
Highest Educational Level														
None	52.0	[44.7,59.2]	8.2	[5.0,13.2]	39.8	[32.5,47.7]	0.011	38.9	[32.7,45.6]	23.8	[18.4,30.2]	37.3	[30.9,44.2]	0.116
Primary	52.9	[48.6,57.2]	6.6	[5.0,8.7]	40.5	[36.5,44.7]		41.4	[38.1,44.8]	17.9	[15.7,20.3]	40.7	[37.3,44.1]	
Secondary +	47.4	[40.4,54.5]	14.9	[10.5,20.7]	37.6	[30.8,45.0]		44.2	[39.9,48.7]	19.5	[16.6,22.8]	36.3	[32.7,40.0]	
Wealth Index														
Poor	52.3	[48.3,56.3]	6.5	[5.0,8.5]	41.1	[37.3,45.0]	0.039	44.1	[40.6,47.5]	17.2	[14.8,20.0]	38.7	[35.3,42.2]	0.292
Middle	49.5	[41.8,57.2]	11.6	[8.0,16.5]	38.9	[32.0,46.3]		44.5	[39.4,49.7]	18.7	[15.1,22.8]	36.8	[31.8,42.1]	
Rich	53.4	[43.4,63.1]	13.9	[7.8,23.4]	32.8	[24.7,42.0]		39.6	[34.7,44.8]	21.0	[17.6,25.0]	39.4	[35.0,43.8]	
Women’s Employment Status														
Unemployed	50.9	[44.7,57.0]	10.9	[8.0,14.6]	38.3	[33.0,43.9]	0.140	41.1	[37.0,45.4]	18.3	[15.7,21.3]	40.5	[36.7,44.5]	0.294
Employed	52.5	[48.0,57.0]	6.8	[5.1,9.0]	40.7	[36.4,45.1]		43.3	[40.0,46.7]	19.7	[17.2,22.5]	36.9	[33.7,40.2]	
Media Access														
No access	58.0	[52.9,62.8]	5.5	[3.8,7.9]	36.6	[31.9,41.5]	0.001	46.8	[42.8,50.9]	16.5	[13.6,19.8]	36.7	[32.8,40.8]	0.027
Access to media	46.6	[42.0,51.3]	10.6	[8.3,13.5]	42.7	[38.4,47.2]		40.7	[37.4,44.2]	20.0	[17.7,22.5]	39.3	[36.1,42.5]	
ANC visits														
<4	54.5	[50.0,58.8]	7.2	[5.2,10.0]	38.3	[34.2,42.6]	0.143	42.6	[39.0,46.3]	18.6	[16.1,21.4]	38.8	[35.3,42.5]	0.884
4+	48.7	[44.0,53.5]	9.5	[7.3,12.2]	41.8	[37.3,46.5]		42.2	[38.7,45.8]	19.4	[16.9,22.3]	38.4	[35.1,41.7]	
Total	51.8	[48.3,55.3]	8.3	[6.7,10.1]	39.9	[36.7,43.2]		42.4	[39.5,45.3]	19.0	[17.1,21.2]	38.6	[35.9,41.3]	

Among home deliveries, a lower proportion of newborns of mothers with primary education received their first PNC within 48 hours of delivery compared with those newborns of mothers with secondary or more education (8% versus 15%). Among newborns delivered at home, the proportion of receiving first PNC within 48 hours increased as household wealth increased. Newborns in households classified as rich had twice as high the proportion of receiving their first PNC within the first 2 days as newborns in poor households (14% versus 7%).

Also among home deliveries, newborns whose mothers had access to media had about twice the proportion of first PNC being within 48 hours compared to newborns whose mothers had no access to media (11% vs. 6%). Likewise, among newborns delivered in a health facility, those whose mothers had access to media were more likely to receive their first PNC within 48 hours compared to newborns whose mothers did not have access to media (20% versus 17%).

### Adjusted backward stepwise logistic regression results for postnatal care

Table 4 below presents the adjusted odds ratios of the association between PNC coverage and demographic and socioeconomic characteristics for newborns delivered at home and in a health facility, respectively. Among home births, newborns whose place of residence at the time of the survey was rural had 40% lower odds of having received any PNC compared to their urban counterparts. The odds of home-born newborns having a PNC visit were 60% higher for newborns whose mothers were exposed or had access to media compared to those whose mothers had no exposure or access. Among home births, newborns whose mothers had (4+ ANC) visits during pregnancy had 60% higher odds of having a PNC visit compared to newborns whose mothers had fewer than four ANC visits.

**Table 4:** Adjusted backward stepwise regression results on the demographic and socioeconomic factors associated with receipt of any postnatal care among the most recent newborns by place of delivery, 2013-14 ZDHS

Variable	Home Births	
	AOR	95% CI
Place of residence		
Urban	1	
Rural	0.6***	0.4 - 0.8
Media exposure		
No exposure	1	
Exposed	1.6***	1.3 - 2.0
No. of ANC visits		
< 4 visits	1	
≥4 visits	1.4***	1.1 - 1.7
Facility Births		
Place of residence		
Urban	1	
Rural	0.8***	0.7 - 0.9
Media exposure		
No exposure	1	
Exposed	1.3***	1.1 - 1.6
Educational Attainment		
None	1	
Secondary or Higher	0.7***	0.5 - 0.9

***p < 0.05

Among newborns delivered in a health facility, and whose place of residence was rural had 20% lower odds of having any PNC compared to those born in urban areas. On the other hand, newborns whose mothers had access or were exposed to media had 70% higher odds of receiving any PNC compared to those whose mothers had none. However, the odds of having any PNC were 30%, lower, for newborns whose mothers had secondary or higher education compared to those whose mothers had no education at all.

### Multinomial backward stepwise regression results for the timing of the first postnatal check-up

Table 5 below presents the adjusted relative risk ratios of the association between timing of first PNC and demographic and socioeconomic characteristics. Separate models were run for home deliveries and deliveries in a health facility. The results show that among home deliveries, the risk of attending first PNC within 48 hours after delivery relative to not attending PNC for newborns whose mothers were exposed or had access to media were 100% higher than those whose mothers were not exposed or had access. Similarly, newborns to mothers who had secondary or higher education had 100% higher odds than that of newborns whose mothers had no education at all. The relative risk for attending first PNC within 48 hours after birth relative to not attending PNC was 90% higher for newborns whose mothers had (4+ ANC) visits than those whose mothers ANC visits were less than four.

**Table 5:** Adjusted relative risk ratios (RRR) results on the demographic and socioeconomic factors associated with timing of the first postnatal checkups among the most recent newborns by place of delivery, 2013-14 ZDHS

Variable	Home Births			
	Within 48 hours		After 48 hours	
	RRR	95% CI	RRR	95% CI
Place of residence				
Urban	1		1	
Rural	0.6	0.4 - 1.1	0.6***	0.4 - 0.8
Media exposure>				
No exposure	1		1	
Exposed	2.0***	1.3 - 3.2	1.5***	1.2 - 1.9
Educational Attainment				
None	1		1	
Secondary or Higher	2.0***	1.2 - 3.1	0.9	0.7 - 1.3
No. of ANC visits				
< 4 visits				
>= 4 visits	1.9***	1.2 - 2.9	1.3***	1.0 - 1.6
Facility Births				
Childs size at birth				
Small	1		1	
Average	0.8***	0.7 - 1.0	0.8***	0.7 - 1.0
Place of residence				
Urban	1		1	
Rural	1.0	0.8 - 1.3	1.3***	1.1 - 1.5
Media exposure				
No exposure	1		1	
Exposed	1.2	1.0 - 1.6	0.8***	0.7 - 0.9
Educational Attainment				
None	1		1	
Secondary or Higher	1.0	0.8 - 1.2	1.2***	1.0 - 1.4

***p<0.05

Table 5 further shows that among newborns delivered at home the relative risk for attending first PNC after 48 hours after delivery to not attending PNC for newborns whose place of residence was rural was 40% lower than for newborns whose place or residence was urban. Further, the relative risk for attending first PNC 48 hours after delivery compared with not attending PNC for newborns whose mothers had media exposure was 50% higher for newborns whose mothers had no media exposure. Similarly, for newborns whose mothers made (4+ ANC) visits, the relative risk for attending first PNC 48 hours after delivery versus not attending PNC was 70% higher than for newborns whose mothers made fewer than four ANC visits.

In contrast, among facility-delivered newborns, the relative risk for attending first PNC within 48 hours after delivery relative to not attending PNC was 20% lower, for newborns whose perceived size at birth was average than for newborns whose perceived size was small. Similarly, the relative risk for a newborn attending PNC 48 hours after birth relative to not attending PNC was 20% for newborns whose perceived size at birth by their mothers was average and were exposed to media. In addition, the relative risk of a newborn having PNC 48 hours after birth relative to not attending was 30% and 20% higher for children whose place of residence was urban than those in rural areas and those with secondary or more education than those with no education at all, respectively.

## Discussion, policy implications, conclusion and limitations

### Discussion

The objectives of this study were to assess the demographic and socioeconomic characteristic associated with PNC coverage and also to examine the factors associated with the timing of first PNC among newborns delivered at home and in health facilities. However, it is cardinal to highlight that our study excluded all women who gave birth through caesarean section as these women and children always receive PNC whilst in the hospital as part of the service.

Our study found that, overall, 55% of newborns received PNC. However, substantial variations were observed by place of delivery. Newborns delivered at home were less likely than those delivered at a health facility to receive PNC (48% versus 58%). This finding is supported by similar studies elsewhere [2, 25, 27]. The difference may be attributed to the fact that births delivered in a health facility are attended to by skilled personnel, who may encourage and educate mothers on the importance of PNC. However, since the data in the DHS does not collect data on the actual services received during PNC, more research may be required to understand the factors that motivate women to go and seek postnatal care for themselves and their newborns. In addition, only 16% of the newborns received PNC within the first two days, according to the 2013-14 ZDHS [15]. Disaggregated by place of delivery, our study found that the timing of PNC within the first 48 hours after birth was more than twice as high among newborns delivered in a health facility compared to those delivered at home (19% versus 8%). The PNC may be this low for both home and facility delivered newborns because only the most recent births were included as part of the analysis implying that young mothers probably those who are more educated and informed are having institutional births. In addition, women are having SMAG members residing with them in their communities thereby making it more likely for more and more women opting to deliver from health institutions as opposed to delivering at home.

Newborns delivered at health facilities, whose size at birth was average as perceived by their mothers had lower odds of having PNC visits within the 48 hours after the child’s birth compared with newborns perceived as small. This finding agrees with other studies [8]. The finding is however counterintuitive, because common sense and other studies informs us that small-sized newborns need extra care after birth compared with newborns of average size [28]. Various factors may help to explain this finding; for example, women in rural areas and women with little education may not know the problems that having a newborn with low birth-weight could bring in the immediate or near future and thus do not see the need for the newborn to be taken for PNC. Besides the aforementioned, it may also be possible that access to monetary resources may be difficult thus making it difficult to seek PNC if the facility is located far from their homes. Moreover, referrals in these communities may not be available as the community side of IMCI may not exist in all the areas of Zambia.

Our study further reveals that, residing in rural areas was associated with lower odds of newborns receiving PNC either for home or facility delivered (40% vs 20%). However, for home births, the odds for newborns in urban and rural areas are twice as high to receive PNC within 48 hours after birth (15% vs. 8%). This seems to be a big problem as proximity to the nearest facility and the economic status of the households where these newborns reside has some effect on whether they access PNC or not. With regard to timing of PNC, place of residence is an important factor that has been cited as influencing the receipt of PNC for newborns within the appropriate timings. According to a study in Nigeria by [29], newborns in places that were predominantly rural in nature had lower odds of having PNC compared with those in predominantly urban regions. Similarly, in Bangladesh [27] found that the place of residence had a significant relationship with newborns’ receipt of PNC and also the timing of first PNC. These studies agree with the results of our study, which found that newborns in rural areas of Zambia had lower odds of newborns having PNC and the timing of first PNC regardless of whether the births were delivered at home or in a health facility.

Our study shows that newborns, whether delivered at home or in a health facility, were more likely to receive PNC if their mothers had access to media. Access to media by mothers to newborns has been found to have a positive association with the use of PNC services among newborns. For instance, women who have access to media have been found to have increased antenatal care visits, and as such they tend to know the benefits of a child having PNC after birth, as they are aware of what problems they encounter if the child does not receive PNC [8, 30–33]. Moreover, this finding may be attributed to the fact that women who have access to media may be more educated, come from well-to-do families or reside in close proximity to health facilities. As such they are well informed and are able to make decisions on the use of their resources and on what health services they can utilise for their newborns in order to ensure that they grow up healthy. For example, Table 5 above shows, among facility delivered newborns whose mothers had secondary or higher education had 100% higher odds of receiving PNC within the 48 hours of delivery.

Furthermore, this study found that newborns delivered at home, whose mothers had four or more ANC visits had higher odds of having PNC and receiving it within the 48 hours after birth. This finding is similar to results of other studies, where the number of ANC visits is an important determinant of PNC use [8, 29]. The plausible explanation of this finding is that through creation of an integrated service provision for pregnant mothers especially among those with poor socio-economic status, may in both urban and rural areas have some multiplier effect for uptake of postnatal services in the future. by making accessible and available at all times may.

### Policy implications

This study demonstrates that various factors influence the utilisation and the timing of PNC for newborns. These factors established by this study provide pointers to policy relating to child health care. It is important that deliveries take place in health facilities in order to receive attention from qualified medical personnel. This may help reduce the high neonatal mortality rate in the future.

The results show that being born in the rural areas reduces the chances of newborns having PNC. Government and other stakeholder should channel more resources to rural areas by building more hospitals and health centres as well as providing health care personnel in these hospitals.

Media access or exposure plays a crucial role by conveying basic child healthcare information to the mothers. Since this study has found an association between media access or exposure with PNC, child health stakeholders and government can utilize the various medias to sensitize people about the importance of PNC for child and mothers’ survival.

The study also shows that mothers’ attendance of the recommended number of ANC visits plays a fundamental role in ensuring that mothers take their newborns for PNC, especially among those delivered at home. Programmes to enhance utilisation of ANC would prove effective at stimulating the utilisation of PNC.

Finally, more research is required to understand the factors that cause low levels of PNC and influence its timing in certain regions like Luapula, Southern, North Western, and Copperbelt regions and among newborns perceived to be small at birth.

### Conclusions

Based on the nationally representative 2013-14 ZDHS, this study identified differences that exist in the factors associated with receipt of PNC and the timing of first PNC among newborns delivered at home and newborns delivered in a health facility. The study shows that a high proportion of newborns regardless of place of delivery are not having PNC. Among the home births, the study shows that being born in the rural areas reduces the odds of having PNC. In addition, mothers’ exposure or access to media, having four or more ANC visits during pregnancy, increases the odds of having PNC. For facility births, newborns whose place of residence was rural and those whose mothers had secondary or higher education, had lower odds of having any PNC conversely, mothers access or exposure to media had a positive influence on PNC.

With regard to timing of PNC, among the home births, mothers’ media exposure or access to media, having secondary or higher education, and had 4+ ANC visits during pregnancy increases the odds of having PNC within 48 hours. Furthermore, attending the first PNC after 48 hours after delivery was determined by place of residence, media exposure and 4+ ANC. On the other hand, the timing of PNC, among the facility births, was influenced by the perceived size at birth of the newborn.

### Limitations

This study had four major limitations: First, the study was limited to only the variables collected in the ZDHS. Factors that may affect PNC care but are not available in DHS could not be examined. For example, we observed differences in receiving PNC among socio-economic factors but we were not able to explore the factors that account for the differences. Second, not only is receipt of PNC important; so is the content of PNC. However, content could not be assessed because of data unavailability. Third, due to the cross-sectional nature of the survey, it was impossible to infer causality in the associations between the covariates in this study and the outcome variables as such we could only do probabilistic conclusions. Fourth, the dependent outcome was based on recall of past events that happened in the two years’ prior the survey.

## Suggestion for further research

This study has revealed that more research may be needed to:

1. Understand if those using more ANC had more complications, or were more urban, educated or from wealthier households.
2. Quantify and qualify why women with average sized babies and those who use less ANC seek and receive PNC and what exactly would get them to use PNC.
3. Assess the availability and quality of facility based current PNC and what women think about it.
4. Examine what community based services are needed/useful to better link the community home births with PNC, and
5. Develop and pilot before scale-up the mechanisms of implementing IEC related to postnatal care to see if it will increase PNC.

## Abbreviations

ANC: Antenatal Care
AOR: Adjusted Odds Ratios
CI: Confidence Interval
DHS: Demographic Health Survey
EAs: Enumeration Areas
EAs: Expanded Program on Immunisations
EmONC: Emergency Obstetric and Newborn Care
ICMCI: Integrated Community Case Management of Childhood Illnesses
IMCI: Integrated Management of Childhood Illnesses
MCDMCH: Ministry of Community Development Mother and Child Health
MoH: Ministry of Health
NNM: Neonatal Mortality
PNC: Postnatal Care
SEA: Standard Enumeration Areas
SMAGs: Safe Motherhood Action Groups
SSA: Sub-Sahara Africa
RRR: Relative Risk Ratio
UNICEF: United Nations Children’s Fund
WHO: World Health Organisation
ZDHS: Zambia Demographic Health Survey
